# Optimizing Perimortem Cesarean Section Outcomes Using Simulation: A Technical Report

**DOI:** 10.7759/cureus.10588

**Published:** 2020-09-22

**Authors:** Maggie O'Dea, Deanna Murphy, Adam Dubrowski, Peter Rogers

**Affiliations:** 1 Simulation, Memorial University of Newfoundland, St. John's, CAN; 2 Obstetrics and Gynecology, Memorial University of Newfoundland, St. John's, CAN; 3 Health Sciences, Ontario Tech University, Oshawa, CAN; 4 Emergency Medicine, Memorial University of Newfoundland, St. John's, CAN

**Keywords:** perimortem caesarean section, maternal cardiac arrest, simulation, medical training, emergency medicine, pmcs, resuscitative hysterotomy, perimortem caesarean delivery, pmcd, postgraduate medical education

## Abstract

Simulation-based medical education (SBME) is an educational technique that enables participants to experience an immersive representation of a clinical event for the purpose of practice, learning, and evaluation. This experience is intended to improve trainees’ competency and confidence in both procedural tasks, as well as team-based and interpersonal skills when responding to real-world clinical encounters. Moreover, SBME improves procedural exposure and competency in low-frequency, high-stakes clinical procedures without the risk of adverse consequences, error, or patient harm - a priority for physician training at all levels.

This technical report describes a novel bi-phasic maternal cardiac arrest simulation that can be used to teach and train post-graduate year one (PGY1) emergency medicine and obstetrics and gynecology trainees in the use of perimortem cesarean sections (PMCS) prior to in-situ exposure. Using a high-fidelity simulation protocol employing training manikins and 3-D printed models of gravid uteri, this bi-phasic simulation, completed over two sessions, six months apart, will equip trainees with the knowledge, skills, and professionalism behaviors necessary for difficult clinical decisions and time-critical procedures.

## Introduction

Perimortem cesarean section (PMCS) is a rare but potentially life-saving procedure that demands knowledgeable leadership and skillful execution. However, opportunities for PMCS exposure, practice, and skills maintenance for resident physicians are limited. To optimize the efficiency of residents’ critical decision-making and the level of procedural skill in such rare but “must-know” clinical encounters, simulation-based medical education (SBME) can be employed [1].

In situations of maternal cardiac arrest at 23 weeks of gestation and beyond, prompt PMCS is critical to increasing the likelihood of both maternal resuscitation and fetal salvage [2]. Because a gravid (pregnant) uterus compresses aortocaval and vital capacity, removal of the fetus and placenta from the uterus via PMCS aids in maternal resuscitation through a number of mechanisms: it alleviates compression, enabling improved venous return and cardiac output; it reduces oxygen consumption and improves ease of ventilation; and it enables improved efficacy of maternal chest compression [3-4]. Indeed, a review of cases published up to 2010 demonstrated that PMCS led to a maternal survival benefit in 19 of 60 cases (31.7%) and was never considered deleterious to maternal survival [5]. Furthermore, due to a fetus’ limited reserve, PMCS offers the best chance of fetal survival post-maternal code [6].

According to clinical guidelines published by the Society of Obstetricians and Gynaecologists of Canada, PMCS is recommended no later than four minutes following maternal cardiac arrest to aid in maternal resuscitation and fetal salvage [4]. However, this narrow time frame is often missed by even senior physicians due to the acute and complex competing demands in maternal resuscitation efforts [1].

Thus, without immersive learning and practice in the high-stress clinical encounter of maternal arrest, Postgraduate Year One (PGY1) residents are unlikely to possess the necessary knowledge or skills to recognize the potential opportunity for PMCS, to make the clinical decision within the critical time frame, and to perform the uncommon procedure competently [1].

This gap is where SBME has an important role to play. In PMCS management, in particular, SMBE has been shown to significantly improve confidence, knowledge, and performance in the management of maternal arrest [7]. Likewise, simulations of maternal arrest were shown to assist in the development of best practices, protocols, and logistical understanding of effective and efficient response [8]. Even for specialist obstetricians highly trained in maternal care, simulation training resulted in improved decision-making during PMCS and the prevention of unnecessary delays in subsequent scenarios [9]. Hence, SBME has incredible potential to equip trainees at all levels with the necessary knowledge, skills, and professionalism for PMCS success.

This technical report describes a simulation designed to optimize the management of PMCS for PGY1 emergency medicine and obstetrics and gynecology trainees. The objective of this simulation is to assess whether the intervention improves trainees’ knowledge and skills in incorporating PMCS into the clinical management of maternal cardiac arrest.

## Technical report

Case

A 28-year-old individual with a visibly gravid uterus has just arrived at the Emergency Department (ED) of a community hospital via ambulance following a syncopal episode in a grocery store. No historical details are available, and no past medical history, allergies, or medications are known. The trainee is the on-call team lead in the ED. There is an ED nurse and a paramedic present to assist.

As the patient is wheeled on a gurney into the room by the paramedic, they go into cardiac arrest.

Context

The simulation was designed to optimize experiential, conceptual, and physical fidelity of maternal cardiac arrest for PGY1 emergency medicine and obstetrics and gynecology trainees while minimizing costs and optimizing reproducibility. It was run in a university-based simulation lab designed to re-create a typical emergency department setting with corresponding equipment and technology, thereby approximating the visual, tactile, and auditory reality of a clinical PMCS encounter.

This simulation was developed using a modified Context, Input, Process, and Product (CIPP) model, to optimize applicability to various learning environments, educational context, inputs, processes, and expected outcomes [10-11]. The details of the case can be adjusted to accommodate different trainees, number of participants, and financial and technological resources. The case can also be performed in a variety of in-situ settings including hospital wards and clinics.

Inputs

To enhance conceptual fidelity, the simulation was designed by obstetric and emergency medicine physicians to ensure scenario credibility and realism. These specialists also functioned as instructors, observing and grading the simulation, and running the debriefing sessions with trainees. There were two embedded participants in the simulation, an ED nurse and a paramedic, to assist the trainees as needed.

As described above, the biphasic case takes place in a community hospital ED. The physical room setup, detailed in Table 1, utilized a hybrid model, which combined both low and high-fidelity elements to optimize both realism and accessibility. The initial portion of the simulation utilized the Laerdal Resusci Anne^©^ manikin (Laerdal, Stavanger, Norway) with gravid uterus overlay, connected to monitors displaying vitals representing cardiac arrest (e.g. heart rate (HR) = 0; respiratory rate (RR) = 0) [7,12]. Lower technological options, such as computer or tablet screens, could be utilized for this purpose as necessary. For the procedural element of the simulation, a portable 3-D printed model of a gravid uterus in abdomen - heretofore concealed under the gurney - was revealed by the embedded nurse and utilized for tactical, hands-on experiential learning. This low-fidelity model, inspired by Sampson, Renz, and Wagner, was designed and created in collaboration with the MUN Med 3D Printing Lab to be easily adapted and reproduced [6].

**Table 1:** Inputs for Managing Maternal Cardiac Arrest ED = Emergency department; ACLS = Advanced cardiovascular life support Anne^©^ manikin: Laerdal, Stavanger, Norway

Case Setting	Community Hospital ED. The paramedic brings a pregnant patient on a gurney into the room, immediately undergoes cardiac arrest (t = 0:00). No attending specialists available.
Personnel	Embedded participants: ED nurse, paramedic
	Facilitators: Obstetrical specialists +/- emergency medicine specialists
Supplies	Telephone (to call code, mobilize specialist team(s))
	Timer
	Gloves
	Laerdal Resusci Anne© manikin with gravid uterus overlay
	Monitors
Carts	Advanced cardiovascular life support (ACLS) cart
	Procedural cart: scalpel, needle driver, pickup, sutures, suture scissors, pack sponges
	Gravid uterus in abdomen model (concealed)

Process

Due to the rarity of maternal cardiac arrest, it was expected that most trainees would lack the experience and knowledge to recognize the opportunity for PMCS and/or perform within the recommended timeline. Thus, to best enable trainees to learn, practice, and apply the intended knowledge and skills of the SBME, the simulation was executed over two identical sessions, held four to six months apart. This design allowed trainees to experience the stress and emotions of an unexpected maternal arrest in the first formative session, as well as to learn and practice in a safe environment. They were then given the opportunity during the second summative session to draw upon and apply the acquired knowledge, skills, and professionalism in the high-pressure environment. 

Pre-Scenario Briefing

Prior to entering the simulation, instructors led a pre-briefing with trainees, carefully explaining the fiction contract of the simulation, as well as learner psychological and emotional safety. During this briefing, particular emphasis was placed on fostering a safe and engaging learning environment. Once the terms of the simulation were agreed upon, trainees were then provided the simulation case, as described above.

Simulation

**Figure 1:**
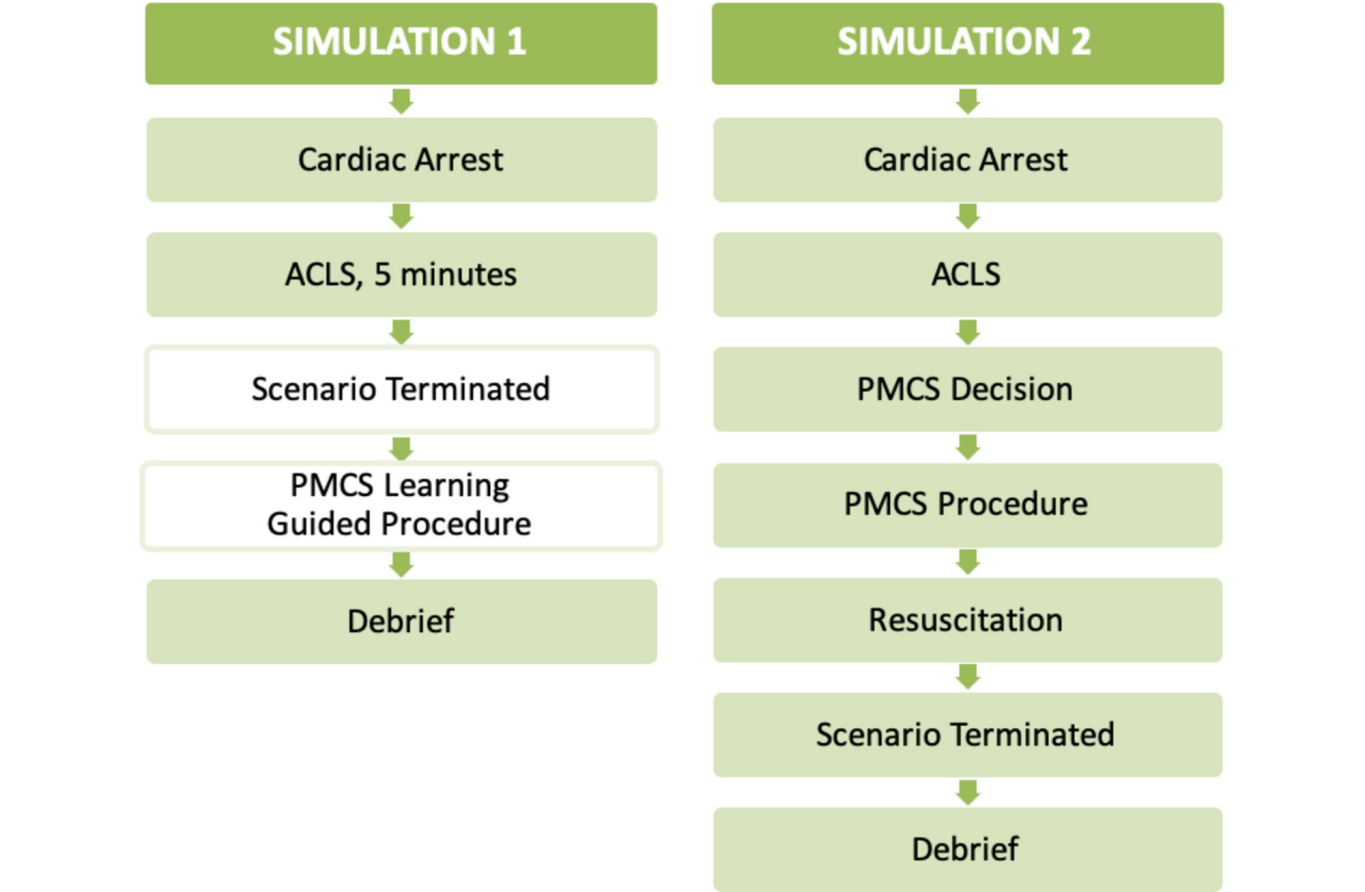
Bi-Phasic Simulation Process Map ACLS = Advanced cardiovascular life support; PMCS = Perimortem cesarean section

The flow of the two simulation sessions (annotated as S1 and S2, respectively), are delineated in Figure 1.

In S1, trainees were presented with a patient with a perceptibly gravid uterus (Laerdal Resusci Anne^©^ manikin with gravid uterus overlay), who went into cardiac arrest upon arrival in the ED. The beginning of the scenario marked time 0:00 of the arrest.

The scenario proceeded with trainees recognizing and responding to maternal cardiac arrest, ideally demonstrating competence in the specifics of Advanced Cardiac Life Support (ACLS) approach in pregnancy (i.e. maintaining manual left uterine displacement). However, in this first iteration, it was not expected that trainees would have the knowledge of PMCS to proceed accordingly in the management. Hence, if, after five minutes of resuscitative efforts with no return of spontaneous circulation (ROSC), there was no initiation of PMCS, the scenario was terminated and the observing instructors revealed themselves. In the circumstance that trainees initiated or attempted to initiate PMCS, the scenario would then be terminated after completion of attempted PMCS (see Simulation 2 for the process map including PMCS).

**Figure 2:**
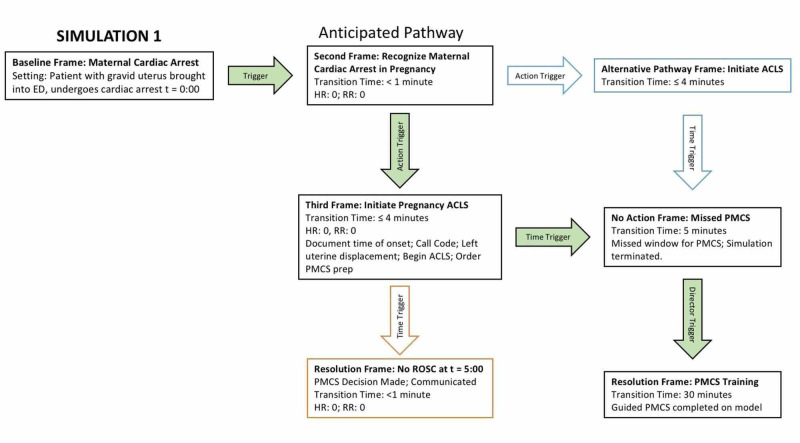
S1 Storyboard ED = Emergency department; ACLS = Advanced cardiac life support; PMCS = Perimortem cesarean section; ROSC = Return of spontaneous circulation

A 30-minute educational session was then conducted in the simulation room, which focused on the three key learning objectives of the simulation: knowledge acquisition, skill acquisition, and professionalism. First, trainees were prompted for PMCS knowledge, followed by instructor-led evidence-based teaching on the management of maternal cardiac arrest, including a detailed explanation of the window of opportunity for PMCS decision-making and initiation [4]. Teaching also incorporated a dedicated discussion of the importance of professionalism during the situation of maternal arrest, including leadership, communication, and emotional regulation. Trainees were then provided the cart containing the low-fidelity model of gravid uterus in abdomen and guided through the complete PMCS procedure. During this time, trainees were encouraged to ask questions and seek clarification to enable trainee-instructor dialogue. Upon completion of the PMCS procedure, S1 was concluded, and trainees proceeded to debrief (detailed below). The intended flow of S1 is illustrated in Figure 2, S1 Storyboard.

S2 occurred four to six months later. Building upon the didactic teaching and tactical experience imparted in S1, trainees were expected to have improved awareness of the need for time-control and communication, as well as the procedural elements of PMCS initiation and completion. However, S2 incorporated three possible prompts for trainees, via the embedded nurse, to ensure that they were given the opportunity to proceed through the scenario productively and apply the knowledge and skills from S1.

The scenario began and proceeded identically to S1. The first prompt occurred at the beginning of ACLS initiation: if the trainees did not verbalize or request timing documentation, the nurse would inquire if they wanted the time recorded and/or for the team to prepare for PMCS *(*i.e. gather the procedural cart).

The second prompt occurred at t = 4:00 as a verbalized timing reminder if trainees hadn't requested a timing reminder. If/when trainees communicated the decision to perform PMCS, the nurse retrieved the gravid uterus in abdomen model, concealed under the gurney, to be used for the surgical procedure.

The third prompt(s) occurred during the PMCS procedure, to provide direction, as needed, to enable the trainees to complete the procedure. This could occur during the removal of the fetus from the uterus, the hand-off of the fetus (to the paramedic, for separate resuscitative efforts), and/or the re-establishment of maternal resuscitation.

**Figure 3:**
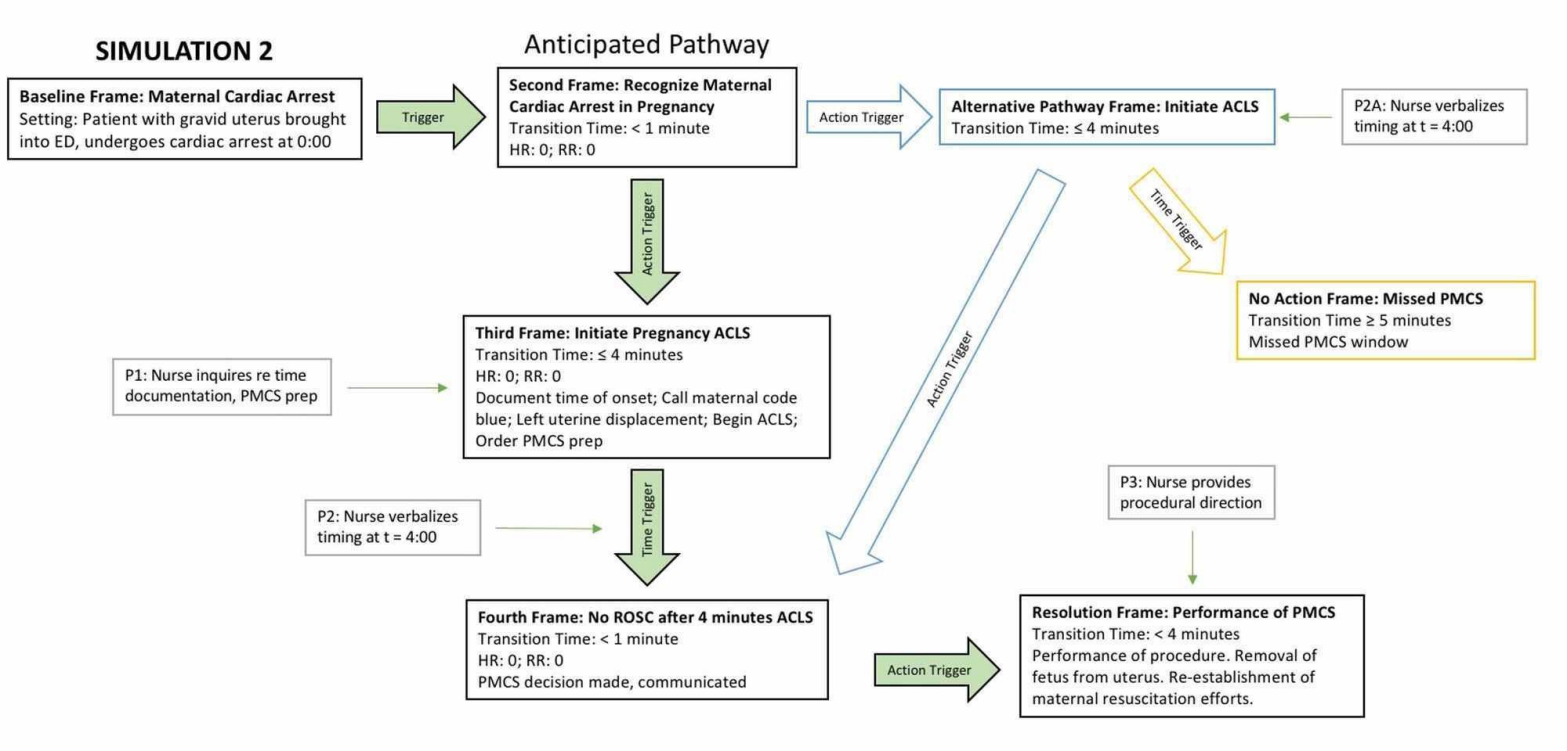
S2 Storyboard ED = Emergency department; ACLS = Advanced cardiac life support; PMCS = Perimortem cesarean section; ROSC = Return of spontaneous circulation

S2 was concluded upon the completion of PMCS and re-establishment of maternal resuscitation for one minute. The flow of S2, including placement for possible prompts, is demonstrated in Figure 3, S2 Storyboard.

This progression through the simulations can be further delineated via a case progression table, as illustrated in Table 2.

**Table 2:** Simulation Progression ACLS = Advanced cardiac life support; PMCS = Perimortem cesarean section; LUD = Left uterine displacement; AED = Automated external defibrillator; ROSC = Return of spontaneous circulation

CASE PROGRESSION	
EXPECTED ACTION 1: Primary Assessment, Recognition of Maternal Cardiac Arrest, Initiation of ACLS for Pregnant Patient	
	Recognizes cardiac arrest
	Calls maternal code to summon a multi-disciplinary team
	Verbally documents the time of arrest
	Requests preparation for PMCS (gathering of procedure cart, supplies)
	Positions patient for high-quality chest compressions in pregnancy, ensuring firm backboard, patient supine with hands in the center of the chest, and directs manual left uterine displacement (LUD)
	Performs or directs the application of Automated External Defibrillator (AED)
	Performs or directs initiation of chest compression and ventilation with continuous LUD
	Performs or directs airway management with head tilt-chin lift maneuver, two-handed bag-mask ventilation with 100% oxygen at 15 L/min and greater
NOTE: if time documentation, PMCS prep not completed, the nurse will prompt in S2	
If completed, proceed to EXPECTED ACTION 2.	
If not completed, proceed to END SCENARIO 2.	
EXPECTED ACTION 2: Decision to Perform PMCS <5 Minutes Post-Cardiac Arrest with no ROSC	
	Communicates decision to perform PMCS to team <5 minutes post-cardiac arrest
	Communicates plan and preparation for PMCS to team
	Organizes equipment
NOTE: Nurse will prompt with timing post-cardiac arrest at 4:00 in S2	
If completed, proceed to EXPECTED ACTION 3	
If not completed, proceed to END SCENARIO 2	
EXPECTED ACTION 3: Initiation of PMCS Within 5 Minutes of Cardiac Arrest, then Re-establishment of Maternal Resuscitation	
	Delegates resuscitation efforts (including LUD) to a team member
	Initiates procedure at the site of resuscitation (does not attempt transport)
	Uses abbreviated antiseptic pour or eliminates antiseptic procedures
	Initiates PMCS: makes midline vertical abdominal incision, from the xiphoid process to the symphysis pubis; separates rectus muscles down midline; opens peritoneum; makes an incision at the lower end of the uterus above the reflection of the bladder; lifts the wall of the uterus from the fetus; cuts the uterus to the fundus; removes the fetus from the uterus, clamps and cuts the cord; hands off the fetus for neonatal resuscitation
	Finalizes PMCS: removes placenta; wipes uterus clean; closes the uterine incision with a running locking stitch of an absorbable suture; closes the abdomen; places a Foley catheter
	Re-establishes maternal resuscitation efforts (LUD, chest compressions, airway management)
NOTE: The nurse will provide directional prompting during the PMCS procedure until the re-establishment of resuscitation.	
If completed, proceed to END SCENARIO 1	
If not completed, proceed to END SCENARIO 2	
END SCENARIO 1: Successful Completion of PMCS	
Simulation terminated by facilitators upon completion of PMCS and re-establishment of maternal resuscitation efforts for one minute. Trainees proceed to debrief.	
END SCENARIO 2: Missed PMCS; Teaching and Training Provided	
Simulation arrested by facilitators, who prompt trainees for PMCS knowledge and understanding and explain missed windows of opportunity for PMCS decision-making and initiation. Trainees are provided a cart containing low-fidelity model of gravid uterus in abdomen, guided through the complete PMCS procedure. Upon finalization of PMCS, the simulation is concluded, and trainees proceed to debrief.	

Post-Scenario Debriefing

At the conclusion of both simulations, trainees were provided with a formal debrief with instructors. Instructor-to-trainee ratios were no greater than 1:1 to ensure optimal support for trainees.

In the debriefing sessions, trainees were first prompted to informally express and examine any emotions experienced during or associated with the simulation. This was of particular importance to this simulation, as the tragic nature of PMCS can be emotionally challenging. The debrief then proceeded to a more formal solicitation of discussion around experiences, and the dual identification of any relative gaps in performance, applying Sigalet’s LEARN framework: Learning objectives; Emotions; Actions and Reflection; and Next steps [13]. This involved reviewing the three learning objectives of the simulation (knowledge acquisition, skill acquisition, professionalism, as detailed below), on which trainees were prompted for personal reflection on performance relative to stated objectives and any perceived gaps. Direct feedback was then given to each trainee to acknowledge and close any identified gaps in knowledge/skill/professionalism behavior. Trainees were once again encouraged to express any emotions elicited by this process. Finally, the debrief session concluded with a discussion of (and positive reinforcement for) knowledge, skills, and professionalism behaviors gained by the simulation to enable trainees to feel effective in their role, equipped for S2, and motivated to engage in further simulations [10].

Products

The simulation utilized both formative and summative assessments. Each simulation was scored by a minimum of two instructors, each of whom scored separately. Each simulation for each participant was recorded to allow for review as needed.

The formative assessment metrics were designed to capture the objective measures of the trainees’ competency in managing maternal cardiac arrest, incorporating elements of knowledge, skill, and professionalism. These were assessed using the Simulation Module for Assessment of Resident Targeted Event Responses (SMARTER) approach to an event-based checklist, to judge whether trainees exhibited the desired standard of care behaviors throughout the simulation [14]. The checklist, illustrated in Table 3, was created to reflect the American Heart Association's scientific statement on maternal resuscitation [5]. Due to the scarcity of validated rating scales for the assessment of obstetrical emergency skills available in the literature, such a formative assessment enabled a more thorough, structured, and objective assessments of the component skills of the complex situation [2,5].

**Table 3:** Assessment of Competencies ACLS = Advanced cardiovascular life support; AED = Automated external defibrillator; LUD = Left uterine displacement

EVENT	CRITICAL RESPONSE	HITS	IG (S2)
Pregnant Patient Undergoes Cardiac Arrest as Entering Emergency Department	Recognized cardiac arrest of a pregnant patient		--
	Called maternal code to summon expert multi-disciplinary team		--
	Verbally documented time of arrest		
	Requested preparation for PMCS – gathering supplies (procedural cart)		
	Initiated ACLS, noting modifications for pregnancy – ensuring firm backboard, patient supine with hands in center of chest, and manual left uterine displacement (LUD)		--
	Began or directed chest compressions		--
	Performed or directed application of AED		--
	Performed or directed airway management with head tilt-chin lift maneuver, two-handed bag-mask ventilation with 100% oxygen at 15 L/min and greater		--
	Remained calm and professional		--
No ROSC After Four (4) Minutes of Resuscitative Efforts	Communicated decision to perform PMCS to team <5 minutes post-cardiac arrest		
	Communicated plan and preparation to team		--
	Organized equipment		
	Remained calm and professional		--
PMCS Procedure	Delegated resuscitation efforts (including LUD) to team members until fetus delivered		
	Initiated procedure at site of resuscitation (did not transport to operating room)		
	Did not wait for surgical equipment beyond scalpel and gloves to begin procedure		
	Used abbreviated antiseptic pour or eliminated antiseptic procedures		
	Made midline vertical abdominal incision from xiphoid process to symphysis pubis		
	Separated rectus muscles down midline		
	Opened peritoneum		
	Made incision at lower end of uterus above reflection of bladder		
	Lifted wall of uterus from fetus		
	Cut uterus to fundus		
	Removed fetus from uterus, clamped and cut cord		
	Handed off fetus for neonatal resuscitation		
	Removed the placenta		
	Wiped uterus clean		
	Closed uterine incision with a running locking stitch of absorbable suture		
	Closed abdomen		
	Placed Foley catheter		
	Re-established maternal resuscitation efforts (LUD, chest compressions, airway management)		
	Worked efficiently and quickly		--
	Remained calm and professional		--

Using an expert panel of four obstetrical and emergency medicine specialists to observe and assess trainees’ performance, the outcomes were graded according to the SMARTER scale of “HITS”: 1 = Observed/performed correctly; 0 = Omitted/failed to perform correctly; X = No opportunity to perform/not required [14]. Further clarification of the level of support and guidance provided during S2 was captured with the Instructor Guidance (IG) column, which detailed whether prompting was given: 1 = No prompting/guidance, 0 = Prompting and/or guidance given to trainee.

Summative evaluation metrics were developed by integrating the assessment of clinical performance (Table 3) with models of outcome-based evaluation such as that used by the National League of Nurses Student Satisfaction and Self-Confidence in Learning Scale [14-15]. These objective metrics, outlined in Table 4, were designed to capture multi-faceted elements of this high-pressure procedure. Namely, the degree to which the simulation improved trainees’ procedural competence and clinical acumen using three main objectives: skills acquisition, knowledge acquisition, and professionalism. The use and comparison of such predefined checklists of target clinical responses enabled an analysis of the trainees' ability to learn and perform unfamiliar medical procedures, as well as whether the SBME objectives were met, and to what degree, across trainees [12].

**Table 4:** Final Evaluation Metrics, S1 and S2 PMCS = Perimortem cesarean section

EVALUATION METRICS	
OBJECTIVE 1: SKILLS ACQUISITION	
By the completion of the simulation, trainees will have demonstrated improved clinical skills in PMCS decision-making, initiation, and completion:	
Followed appropriate guidelines for maternal resuscitation
Made informed decision to initiate PMCS intervention within the recommended time period
Performed all PMCS procedures competently
Demonstrated application and mastering of simulation content
OBJECTIVE 2: KNOWLEDGE ACQUISITION	
By the completion of the simulation, trainees will have demonstrated improved knowledge of the standard of care for a pregnant patient presenting with sudden cardiac arrest:	
Grasped importance of the decision for PMCS
Recognized importance of timing of PMCS timing
Understood guidelines for PMCS
Understood guidelines for maternal resuscitation
Demonstrated knowledge of PMCS preparation and progression
OBJECTIVE 3: PROFESSIONALISM	
By the completion of the simulation, trainees will have garnered and successfully applied skills in efficient clinical decision-making and interprofessional communication in a high stress, time-sensitive clinical scenarios:	
Assumed leadership role
Communicated decisions clearly
Remained calm and professional in a stressful situation
Effectively managed interdisciplinary team and resources

## Discussion

The use of simulation-based medical education enables trainees to experience an immersive representation of a clinical event for the purpose of learning without the risk of adverse consequences or patient harm [11]. Its role is particularly significant in high acuity, low-frequency clinical presentations for which medical practitioners and trainees alike have limited hands-on exposure.

PMCS is one such time-critical and life-saving procedure that demands competent and confident execution. However, opportunities for exposure to this rare but ‘must know’ clinical encounter for residents and physicians alike are extremely limited. Without immersive learning and practice in maternal cardiac arrest, resident physicians are unlikely to possess the knowledge or skills to recognize the potential opportunity for PMCS, make the clinical decision within the recommended time frame, and perform the rare procedure effectively [12]. Hence, SBME can be utilized to improve trainees’ competency and confidence in future clinical encounters of maternal arrest [6].

This technical report describes a novel bi-phasic simulation that can be used to equip PGY1 emergency medicine and obstetrics and gynecology trainees with the necessary knowledge, skills, and professionalism behaviors to manage maternal cardiac arrest and appropriately initiate PMCS within recommended guidelines. In the application and design of this simulation case, we sought to reduce barriers to implementation by creating 3D printed low fidelity models of gravid uteri in abdomen that are both cost-effective and attainable. This further enables accessibility, particularly in resource-limited training centers without access to high-fidelity models.

Ultimately, the goal of this simulation was to enable practitioners to be better equipped to respond to maternal arrest crises, and appropriately and competently carry out PMCS when indicated, within recommended timelines. Following its completion, trainees exhibited improved competency and confidence in both procedural tasks as well as team-based skills when responding to a clinical encounter of maternal cardiac arrest. It can be expected that trainees of this simulation will be better equipped to respond effectively in any such high-stakes, real-world clinical encounters [16].

## Conclusions

Maternal cardiac arrest - and the timely implementation of PMCS - is a rare and emotionally challenging clinical situation that has the potential to save lives. Any opportunity for exposure, training, and the application of knowledge, skills, and professionalism behaviors in a controlled environment is a valuable use of resources for post-graduate medical education programs. This technical report describes the design and implementation of a novel bi-phasic simulation of maternal cardiac arrest for PGY1 emergency medicine and obstetrics and gynecology trainees. Future efforts will be made to enhance the surgical tactile realism of the 3D-printed gravid uterus in abdomen model, as well as its reproducibility, to optimize its potential use in SBME programs across Canada and around the world.
